# Effects of Game-Related Tasks for the Diagnosis and Classification of Gaming Disorder

**DOI:** 10.3390/bios14010042

**Published:** 2024-01-13

**Authors:** Jeongbong Choi, Youngseok Choi, Young-Chul Jung, Jeyeon Lee, Jongshill Lee, Eunkyoung Park, In Young Kim

**Affiliations:** 1 Department of Biomedical Engineering, Hanyang University, Seoul 04763, Republic of Korea; 2 Department of Electronic Engineering, Hanyang University, Seoul 04763, Republic of Korea; 3 Department of Psychiatry, Yonsei University College of Medicine, Seoul 04763, Republic of Korea; 4 Department of Biomedical Engineering, Soonchunhyang University, Asan 31538, Republic of Korea

**Keywords:** gaming disorder, EEG, HRV, task dependence, self-regulation, video game

## Abstract

Gaming disorder (GD) is an addictive behavior characterized by an insatiable need to play video games and shares similar symptoms with the failure of self-control due to a decline in cognitive function. Current GD diagnostic and screening tools rely on questionnaires and behavioral observations related to cognitive functions to assess an individual’s capacity to maintain self-control in everyday life. However, current GD screening approaches rely on subjective symptoms, and a reliable diagnosis requires long-term clinical follow-up. Recent studies have measured biosignals along with cognitive functional tasks to provide objectivity to GD diagnosis and to acquire immediate results. However, people with GD are hypersensitive to game-related cues, so their responses may vary depending on the type of stimuli, and the difference in response to stimuli might manifest as a difference in the degree of change in the biosignal. Therefore, it is critical to choose the correct stimulus type when performing GD diagnostic tasks. In this study, we investigated the task dependence of cognitive decline in GD by comparing two cognitive functional tasks: a continuous performance task (CPT) and video game play. For this study, 69 young male adults were classified into either the gaming disorder group (GD, *n* = 39) or a healthy control group (HC, *n* = 30). CPT score, EEG signal (theta, alpha, and beta), and HRV-HF power were assessed. We observed differences in the left frontal region (LF) of the brain between the GD and HC groups during online video game play. The GD group also showed a significant difference in HF power of HRV between CPT and online video gaming. Furthermore, LF and HRV-HF significantly correlated with Young’s Internet Addiction Test (Y-IAT) score, which is positively associated with impulsivity score. The amount of change in theta band activity in LF and HRV-HF—both biomarkers for changes in cognitive function—during online video game play suggests that people with GD express task-dependent cognitive decline compared with HC. Our results demonstrate the feasibility of quantifying individual self-regulation ability for gaming and underscore its importance for GD classification.

## 1. Introduction

Gaming disorder (GD) is an addictive behavior characterized by an insatiable need to play video games [1,2,3]. People with GD prioritize games over important goals in their daily lives and constantly face negative consequences because their goal-directed behavior is disturbed by game-related activity [4,5]. Over the last ten years, the exponential growth of the video game industry and the COVID-19 pandemic have increased the global prevalence of gaming addiction [6].

GD shares similar symptoms with the failure of self-control due to a decline in cognitive function [7,8,9]. Self-control refers to the ability to maintain control over one’s behavior to pursue priority goals, whereas people who lack self-control exhibit impulsive behavior and erratic decision-making [10]. Current GD diagnostic tools rely on questionnaires and behavioral observations related to cognitive function to assess an individual’s capacity to maintain self-control in everyday life [11].

Accordingly, current GD screening approaches rely on subjective symptoms; however, a reliable diagnosis based on such symptoms requires more than one year of clinical follow-up [12]. Therefore, recent studies have measured biosignals along with cognitive functional tasks to bring objectivity to GD diagnosis and to acquire immediate results [13,14].

The type of stimulus may be an important factor for evoking a GD-related response because people with GD are hypersensitive to game-related cues [15,16,17,18]. Because the difference in response to stimuli might manifest as a difference in the degree of change in the biosignal, it is critical to choose the correct stimulus type when performing GD diagnostic tasks [19]. Existing cognitive functional tasks are insufficient for assessing GD because they are unrelated to video games, in which GD patients exhibit compulsive behaviors. A previous study found no difference between normal and GD groups when measuring heart rate variability (HRV) during a general cognitive functional task; however, a difference was observed during video game play [20]. This suggests that no difference may be observed if an appropriate stimulus is not presented.

Therefore, in this study, we investigated the task dependence of cognitive decline in people with GD. We compared two cognitive functional tasks: a continuous performance task (CPT) and video game play [21,22]. We used a specific video game in which GD patients exhibited compulsive behaviors to enhance the cue reactivity, and we used the CPT as the general cognitive functional task not related to video game play. The biosignals were measured from participants while performing the CPT and video game play. From the measured biosignals, biomarkers related to cognitive function were extracted for each task, and a comparison was performed between two groups: general game users and game users with GD. Biomarkers showing differences between groups were further analyzed. In more detail, correlations between GD severity and difference in response according to the task were investigated to delineate the task-dependent response of cognitive decline in GD.

## 2. Materials and Methods

### 2.1. Subjects

Sixty-nine young male adults were recruited for this experiment (mean ± standard deviation; 22.0 ± 2.8 years). Because males typically present with gaming disorders more frequently than females [23], we only considered male adults to reduce gender differences as much as possible. Regarding educational level, most participants (*n* = 57) were enrolled in college. Seven had graduated from college, four had graduated from high school, and one had graduated from middle school. All participants were skilled players of the online video game “League of Legends (LoL)” (Riot Games, 2009). Participants underwent GD diagnosis using Young’s Internet Addiction Test (Y-IAT) and the Korea Internet Addiction Test (K-scale). Participants with a severity score > 50 in Y-IAT were classified into the gaming disorder group (GD, *n* = 39), and participants with a Y-IAT score ≤ 50 were classified into the healthy control group (HC, *n* = 30) [24,25,26]. A certified psychiatrist performed an interview with the GD group using criteria from the Diagnostic and Statistical Manual of Mental Disorders (DSM-5) [27,28,29] to identify the existence of any co-morbidities. Depression (Beck Depression Inventory), tension (Beck Anxiety Inventory), alcohol use disorder, attention deficit disorder (Wender Utah Rating Scale), and impulsiveness (Barratt Impulsiveness Scale) were also evaluated [30,31,32,33,34]. Any participant with a neurological or cardiovascular disease or who was taking psychiatric medications was excluded from the study. Informed consent was collected from all subjects, and the Institutional Review Board approved the protocol (HYI-16-044).

### 2.2. Procedure

**Figure 1:**
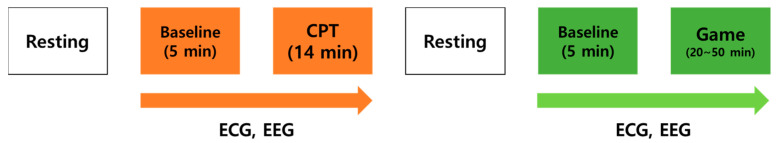
Experimental procedure.

After screening at the hospital by a certified psychiatrist, the experimental procedure including the measurement of biosignals was carried out in a laboratory-based environment. Electrodes for recording electroencephalograms (EEGs) and electrocardiograms (ECGs) were attached to the participant while sitting in a chair. An five-minute baseline EEG and ECG were recorded with the participant in a resting state after sufficient rest for the CPT session (Figure 1). After baseline measurements were performed, EEGs and ECGs were recorded while the participant was performing a 14 min CPT [35] consisting of 18 blocks of 20 trials per block. In a total of 360 trials, one letter was randomly presented for each trial, and the subject was instructed to respond by pressing the corresponding key for the letter as quickly as possible—except for the letter X [36]. After completing the CPT, participants had sufficient rest, and the baseline was measured before playing the online video game. Riot’s “League of Legends (LoL)” was used for the game task, and the participant’s EEGs and ECGs were recorded during game play (approximately 20–50 min).

### 2.3. Data Acquisition and Preprocessing

We simultaneously recorded EEGs and ECGs for each participant. EEGs were collected using a Waveguard 64 EEG sensor-cap (ANT-Neuro, Enscheda, The Netherlands). Electrical signals were collected while maintaining the impedance of electrodes placed at the 10-10 montage below 10 kilo ohms, and the collected electrical signals were stored at a sampling frequency of 1024 Hz in the BRAINBOX EEG-1166 system (Braintronics, Almere, The Netherlands). The measurement system used FPz and AFz as the active G1/G2 ground to eliminate common mode interference, and the reference signal was measured with the electrode attached to the earlobe. To remove noise from the measured signal, a notch filter with 60 Hz center frequency and high-pass and low-pass filters with 1 Hz and 30 Hz cutoff frequencies, respectively, were applied. After removing eye blink noise from the filtered signal, it was expressed in a time-frequency domain through Morlet wavelet transformation.

ECG recordings were collected with a sampling frequency of 200 Hz using a 3-lead monitoring system (MP150 BIOPAC Systems, Santa Barbara, CA, USA). For noise removal, a high-pass filter with a 0.1 Hz cutoff frequency, a low-pass filter with a 15 Hz cutoff frequency, and a notch filter with a 60 Hz center frequency were applied. The R peak was extracted from the measured signal using the Pan and Tompkins algorithm, and the RR interval signal was extracted through resampling using ectopic removal and cubic spline interpolation [37,38]. We used customized MATLAB algorithms to process signals including the EEGs and ECGs (The MathWorks, Inc., Natick, MA, USA).

### 2.4. Data Analysis

For the analyses, indices related to cognitive function were extracted from the EEGs and ECGs for each of four experimental periods: baseline before CPT, during CPT, baseline before online video gaming, and during online video gaming.

The EEG signal was analyzed for power in the theta (4–8 Hz), alpha (8–13 Hz) and beta (13–30 Hz) bands, which are the known frequency bands associated with cognitive function. For CPT and online video gaming, the relative power changes to the corresponding each baseline were calculated for theta, alpha, and beta power for each EEG electrode channel.

The RR interval signal of each ECG was analyzed by converting it into a frequency domain signal representing heart rate variability (HRV) through wavelet transformation [39]. Among the converted signal components, the relative power change compared with the corresponding baseline was calculated for each task for the high-frequency component of the HRV (HRV-HF: 0.15–0.4 Hz) [40], which is known to be related to cognitive function [41].

To analyze the extracted biomarkers, a mixed-model analysis of variance (ANOVA) for groups and tasks was performed on the relative changes in the biomarkers from the EEGs and HRV. GD and HC group comparisons evaluated differences between the two groups, while task comparisons (CPT versus online video game) determined differences within each subject. A post hoc analysis with Bonferroni correction was performed on the biomarkers that differed significantly in comparisons. To confirm task-dependent cognitive decline, a correlation between GD severity and response difference according to task was investigated. Spearman’s rank correlation coefficient was calculated for biomarkers that differed significantly between the GD and HC groups.

## 3. Results

### 3.1. Demographics and Clinical Characteristics

There were no significant differences in age and intelligence level between the GD and HC groups (Table 1). Although the GD group showed a higher average value than HC for indicators of depression, anxiety, alcohol use disorder, and ADHD, there were no significant differences between the two groups. Most of the impulsivity indicators did not show a significant difference between the GD and HC groups, but the GD group had significantly higher attentional impulsivity than the HC (t = 2.523, *p* = 0.014).

### 3.2. CPT Score

**Figure 2:**
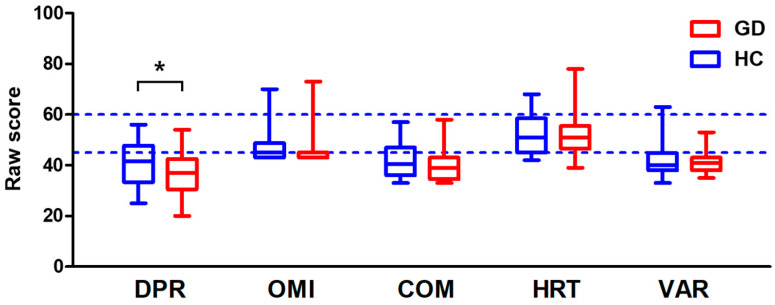
Continuous performance task (CPT) results for the groups of healthy controls (HC) and gaming disorder (GD) participants. Blue indicates the value for healthy controls, and red indicates the value for GD participants. The dotted lines indicate the range of average values; values above the upper dotted line indicate poor performance, and values below the lower dotted line indicate excellent performance. (DPR: detectability; OMI: omission error; COM: commission error; HRT: hit reaction time; VAR: variability) (* *p* < 0.05).

To evaluate the difference in cognitive function between the two groups during CPT, scores were calculated for detectability (DPR), commission error (COM), omission error (OMI), hit reaction time (HRT), and variability (VAR) (Figure 2). DPR indicates the ability to distinguish between targets (any letter except ‘X’) and non-targets (letter ‘X’), and COM and OMI indicate errors caused by a missed target and a response to a non-target, respectively. HRT refers to the average time taken to respond to the target, and VAR is the consistency of HRT between blocks of CPT. A lower score for each item indicates better performance on cognitive tasks. When comparing the GD and HC groups for each CPT item, there were no significant differences except for DPR (DPR, t = 2.144, *p* = 0.035). When the average score of each item was compared with the 45–60-point interval, which corresponds to the average range of a normal person without underlying disease, both the GD and HC groups had the same or lower values than the corresponding interval.

### 3.3. Brain Activity

**Figure 3:**
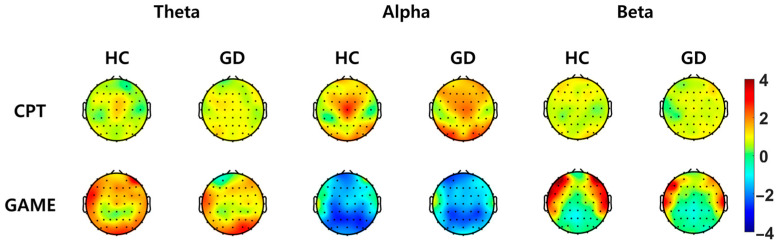
Topographical map for each frequency band of EEG power increase (%) compared with the corresponding baseline for groups of healthy controls (HC) and gaming disorder (GD) participants (**left**: Theta; **middle**: Alpha; **right**: Beta). Red indicates an increase compared with the baseline period, and blue indicates a decrease compared with the baseline period. The upper column shows the amount of increase in EEG power during a continuous performance task (CPT), and the lower column shows the amount of increase in EEG power when playing a game (GAME).

A topographical map showing the average value of power change for each group compared with the corresponding baseline for each channel in the theta, alpha, and beta bands is presented in Figure 3. When looking at the difference in average value according to electrode position, it was possible to confirm the difference between the groups in the frontal area, especially in the left frontal area, during online video game play.

**Figure 4:**
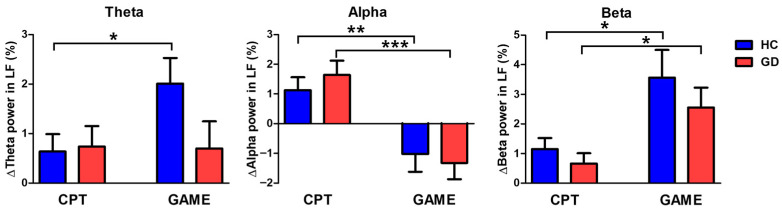
Amount of increase in EEG power compared with baseline by frequency band in the left frontal area for healthy controls (HC) and gaming disorder (GD) participants (%) (**left**: Theta; **middle**: Alpha; **right**: Beta). Blue indicates the value for HC, and red indicates the value for GD. The left side of each graph shows the EEG power increase when performing a continuous performance task (CPT), and the right side shows the EEG power increase when playing a game (GAME). (* *p* < 0.05, ** *p* < 0.005, *** *p* < 0.001).

A mixed-model ANOVA was used to estimate statistical differences according to type of task in the F7, F3, AF7, AF3, and F5 channels corresponding to the left frontal region (Figure 4). The mixed-model ANOVA revealed no significant interaction between the group and task except for the beta band (theta, F [1, 40] = 0.657, *p* = 0.894; alpha, F [1, 40] = 1.385, *p* = 0.172; beta, F [1, 40] = 1.791, *p* = 0.046). The group did not show a statistically significant effect for change in each frequency band power (theta, *p* = 0.799; alpha, *p* = 0.698; beta, *p* = 0.091). The type of task had a statistically significant effect on alpha and beta band power change. Theta band power change was not significantly different between CPT and online video gaming in GD participants (*p* = 0.951). In contrast, there was a significant increase in theta band power among the HC participants during online video game play (*p* = 0.032).

### 3.4. Parasympathetic Activity Comparison

**Figure 5:**
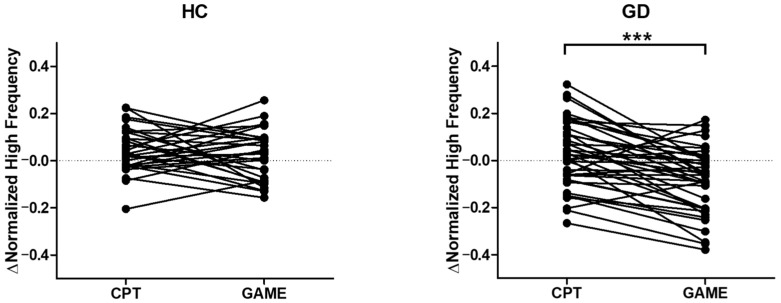
Changes in high-frequency components of heart rate variability (HRV) for each group of healthy controls (HC) and gaming disorder (GD) participants compared with the baseline by task (**left**: healthy controls; **right**: gaming disorder). The left side of each graph shows the HRV-HF increase during a continuous performance task (CPT), and the right side shows the HRV-HF increase when playing a game (GAME). (*** *p* < 0.001).

A mixed-model ANOVA revealed no statistically significant interaction between the group and task in HRV-HF power (F [1, 40] = 1.07, *p* = 0.428). However, a main-effect analysis showed a significant effect of task on HRV-HF power (*p* < 0.001). An additional paired *t*-test analysis (Figure 5) revealed a significant difference in the change in HF power between CPT and online video gaming in GD participants, while there was no significant difference observed in HC participants (GD, *p* < 0.001; HC, *p* = 0.363).

### 3.5. Correlation Analysis

**Figure 6:**
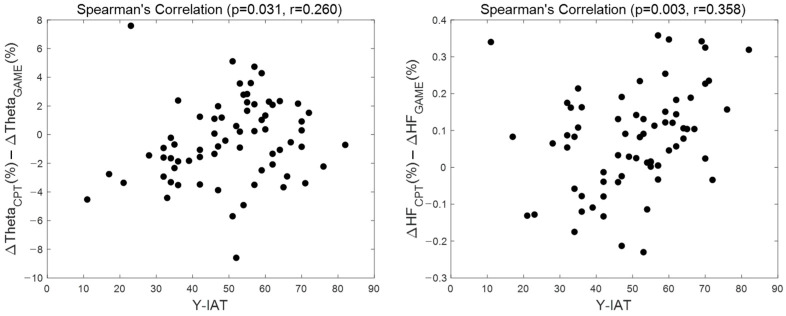
Scatterplot between GD score and biomarkers. The graph on the left shows the correlation between Y-IAT score and the gap of normalized HRV-HF power change between tasks ($\Delta Theta_{CPT}-\Delta Theta_{GAME}$). The graph on the right shows the correlation between Y-IAT score and the gap of theta band power change in left frontal region between tasks ($\Delta HF_{CPT}-\Delta HF_{GAME}$).

Spearman’s correlation analysis was applied to confirm correlations between GD severity and difference in biomarker response according to tasks to quantify the task-dependent response of cognitive decline in GD. Correlation analysis revealed that the response difference in biomarkers between CPT and online video game play had a significant relationship with gaming-disorder severity (Figure 6). The degree of difference in the response of theta band and HRV-HF between CPT and online video gaming was significantly associated with Y-IAT score (theta, *p* = 0.031, r = 0.260; HRV-HF, *p* = 0.002, r = 0.357).

## 4. Discussion

We investigated the task dependence of cognitive decline in people with gaming disorder (GD). In this study, we compared the biosignals measured from participants while conducting a continuous performance task (CPT) or playing an online video game. The CPT was not related to online video gaming, and the online video game used in this study was a game that participants reported enjoying in order to increase their immersion in the task. The potential for comorbidities was eliminated by pre-screening all participants, and this provided an unobstructed observation of GD results in this study. We assumed that the relevance of tasks to online video gaming would affect biosignals associated with cognitive function. We also assumed that changes in biosignals caused by tasks would affect people in the healthy control (HC) and gaming disorder (GD) groups differently.

Our results revealed that biosignals were affected by both the group and task. Theta band activity in the left frontal region (LF) of the brain was significantly different between CPT and online video game play in HCs, while there was no difference in GD participants (Figure 4). The difference in theta band power change between the HC and GD groups for online video game play may represent impaired cognitive control in the GD group. Cognitive control is the ability to present appropriate behavior to achieve goals and suppress habitual responses [42]. Increased theta band power in the frontal area represents increased cognitive control during tasks [43,44]. Therefore, increased theta band power in the LF during online video gaming versus CPT in the HC group indicates that online video game play requires more cognitive control than CPT. In contrast, the difference in the amount of LF theta power increase between HC and GD during online video game play—i.e., a lower theta power increase in the GD group compared with the HC group—may indicate a loss of cognitive control in the GD participants due to a deterioration of prefrontal function [45,46].

Although alpha and beta activity in LF were not significantly different between groups, they showed task-dependent changes (Figure 4). Alpha activity in LF is associated with working memory, which links the task and the corresponding behavior. Specifically, decreases in alpha activity are associated with actual responses related to the working memory [47,48]. Therefore, the increase in alpha activity during CPT but the decrease during online video game play in both the HC and GD groups could mean that the online video game required greater utilization of working memory than CPT. Combined with the theta result, low alpha and theta levels in the frontal area during task performance implies that the participants were using their working memory but were in a state of boredom while habitually performing tasks [49]. The beta activity in the LF indicates impulsivity control, and our result show that online video gaming requires greater inhibition of impulsive behaviors than CPT [46,50].

The task dependency of cognitive decline in GD participants is also supported by heart rate variability (HRV) (Figure 5). The amount of change in the high-frequency component of HRV (HRV-HF) was significantly different between CPT and online video game play in GD participants, while there was no difference in HCs. HRV-HF represents parasympathetic activity by the vagal nerve, which is related to prefrontal activity [51,52,53,54]. The difference in HRV between the HC and GD groups, which appears only during online video gaming, could be interpreted as task-dependent cognitive decline in GD [13,20].

We further found that the differences in response between CPT and online video game play for theta activity of the LF and HRV-HF were significantly correlated with GD severity score, which was positively correlated with impulsivity score (Figure 6). These correlations suggest that theta activity in the LF and HRV-HF are associated with impulsivity, which is a symptom of reduced cognitive control [55].

This study has several limitations. First, this study only included male participants. While this decision was made considering the prevailing male predominance in gaming disorder, it is also important to recognize the existence of female gaming disorder patients with potentially distinct clinical characteristics. Including both genders in future studies would contribute to a more comprehensive understanding of gaming disorder. Second, the evaluation of gaming disorder in this study utilized a non-specific tool (Young’s Internet Addiction Test, Y-IAT), employing a scale for overall online activity rather than a dedicated gaming assessment. However, the recruited participants were experienced in and familiar with the same game, and they were instructed that the primary focus of the Y-IAT questionnaire in this study was Internet gaming. Lastly, due to the limited exploration of participants’ psychological profiles in our study, we missed the opportunity to investigate the nuanced relationship between addictive gaming behavior and physiological features, specifically heart rate variability (HRV) and electroencephalograms (EEGs). A more exhaustive examination of psychological factors holds the potential to yield deeper insights into the association between addictive gaming behavior and the manifestations observed in HRV and EEGs.

In this study, the direction of changes in biomarkers, which revealed a substantial difference between HC and GD groups, could be interpreted as a decline in game user self-regulation. Cognitive function sustains a person’s self-regulation and may be affected by changes in the power of the theta, alpha, and beta bands. Failure of self-regulation interferes with goal-directed behavior control and results in stimulus-driven habitual behavior control [56,57]. Patients with GD choose to play a game that can provide immediate pleasure rather than struggling for a goal because habitual behavior control regulates behavior by focusing on quick rewards [5]. Consequently, regaining self-regulation is critical for treating GD [58,59]. Our findings demonstrate the feasibility of quantifying individual self-regulation ability for gaming by employing biometrics measured during online video game play. Thus, the more highly game-related a stimulus is, the more accurate the GD diagnosis will be.

Theta band activity in LF and HRV-HF are biomarkers of changes in cognitive function. The changes in these biomarkers during online video game play were consistent with our hypothesis that people with GD express task-dependent cognitive decline compared with HCs. Therefore, our results underscore the importance of task evaluation for GD classification.

## Figures and Tables

**Table 1:** Characteristics of participants. Data are presented as means (standard deviations). (* *p* < 0.05, ** *p* < 0.005).

	Control (*n* = 30)	Gaming Disorder (*n* = 39)	*p*-Value
Young internet addiction test	36.72 (9.17)	61.41 (7.74)	0.001 **
Korean internet gaming disorder scale	65.91 (12.42)	83.88 (12.53)	0.001 **
Age, years	22.81 (2.46)	23.07 (2.48)	0.660
Full scale intelligence Quotient	111.72 (10.26)	110.57 (14.95)	0.713
Beck depression inventory	7.88 (5.94)	9.76 (7.64)	0.261
Beck anxiety inventory	4.97 (5.63)	7.83 (6.45)	0.054
Barratt impulsiveness scale—non-planning	21.53 (3.15)	22.49 (3.48)	0.235
Barratt impulsiveness scale—motor	14.5 (3.08)	15.05 (3.64)	0.503
Barratt impulsiveness scale—attentional	13.06 (2.87)	14.85 (3.04)	0.014 *
Alcohol use disorder identification test	9.28 (5.3)	10.39 (6.99)	0.465
Conner’s adult hyperactivity restlessness	47.78 (4.21)	48 (5.48)	0.855
Wender Utah rating sale for ADHD	24.72 (17.42)	27.17 (17.68)	0.561

## Data Availability

The data are not publicly available due to the fact that personal information is included. In order to protect the privacy and confidentiality of individuals, access to the data is restricted.
